# In Vitro Macrophage Immunomodulation by Poly(ε-caprolactone) Based-Coated AZ31 Mg Alloy

**DOI:** 10.3390/ijms22020909

**Published:** 2021-01-18

**Authors:** Andreea-Mariana Negrescu, Madalina-Georgiana Necula, Adi Gebaur, Florentina Golgovici, Cristina Nica, Filis Curti, Horia Iovu, Marieta Costache, Anisoara Cimpean

**Affiliations:** 1 Department of Biochemistry and Molecular Biology, Faculty of Biology, University of Bucharest, 91-95 Splaiul Independentei, 050095 Bucharest, Romania; andreea.mariana.negrescu@drd.unibuc.ro (A.-M.N.); madalina.georgiana.necula@drd.unibuc.ro (M.-G.N.); cristina.nica@drd.unibuc.ro (C.N.); marieta.costache@bio.unibuc.ro (M.C.); 2 Advance Polymer Materials Group, Faculty of Applied Chemistry and Materials Science, University Politehnica of Bucharest, Gh. Polizu 17, 011061 Bucharest, Romania; adi.ghebaur@upb.ro (A.G.); filis.curti@chimie.upb.ro (F.C.); horia.iovu@upb.ro (H.I.); 3 Department of General Chemistry, Faculty of Applied Chemistry and Material Science, University Politehnica of Bucharest, Gh. Polizu 1-7, 011061 Bucharest, Romania; florentina.golgovici@upb.ro

**Keywords:** macrophage, inflammatory response, osteoclastogenesis, magnesium alloy, PCL-coating, electrochemical behaviour

## Abstract

Due to its excellent bone-like mechanical properties and non-toxicity, magnesium (Mg) and its alloys have attracted great interest as biomaterials for orthopaedic applications. However, their fast degradation rate in physiological environments leads to an acute inflammatory response, restricting their use as biodegradable metallic implants. Endowing Mg-based biomaterials with immunomodulatory properties can help trigger a desired immune response capable of supporting a favorable healing process. In this study, electrospun poly(ε-caprolactone) (PCL) fibers loaded with coumarin (CM) and/or zinc oxide nanoparticles (ZnO) were used to coat the commercial AZ31 Mg alloy as single and combined formulas, and their effects on the macrophage inflammatory response and osteoclastogenic process were investigated by indirect contact studies. Likewise, the capacity of the analyzed samples to generate reactive oxygen species (ROS) has been investigated. The data obtained by attenuated total reflection Fourier-transform infrared (FTIR-ATR) and X-ray photoelectron spectroscopy (XPS) analyses indicate that AZ31 alloy was perfectly coated with the PCL fibers loaded with CM and ZnO, which had an important influence on tuning the release of the active ingredient. Furthermore, in terms of degradation in phosphate-buffered saline (PBS) solution, the PCL-ZnO- and secondary PCL-CM-ZnO-coated samples exhibited the best corrosion behaviour. The in vitro results showed the PCL-CM-ZnO and, to a lower extent, PCL-ZnO coated sample exhibited the best behaviour in terms of inflammatory response and receptor activator of nuclear factor kappa-B ligand (RANKL)-mediated differentiation of RAW 264.7 macrophages into osteoclasts. Altogether, the results obtained suggest that the coating of Mg alloys with fibrous PCL containing CM and/or ZnO can constitute a feasible strategy for biomedical applications.

## 1. Introduction

Recently, Mg and its alloys have gained more and more attention as potential candidates for orthopaedic applications due to their good biodegradability, biocompatibility and favourable mechanical properties [1,2,3]. However, the main limitation is their low corrosion resistance in physiological environments [4,5,6,7], which may lead to an excessive inflammatory response in the living system. The inflammatory response produced by the implantation of a biomaterial in the host, determines the long-term performance of the implant [8]. Therefore, the interaction between the metallic implant and organism should lead to a beneficial immune reaction [9]. Over the last few decades, in terms of bone biology, a great progress has been made in the understanding of the interplay between bone and immune system, revealing that the immune response plays a key role in bone biomaterial-stimulated osteogenesis [10] and inflammatory fibrous tissue encapsulation [11]. It is well known that the inflammatory reaction involves the activation of a wide range of cell types, including one of the most important effectors of the innate immune response, namely macrophages [11]. Several studies showed that the end results of the inflammatory response and the long-term immune reaction to various biomaterials is dictated by macrophages, therefore turning them into primary targets for immune system modulation. Macrophages, exhibit high plasticity, being capable of switching their phenotype in response to various environmental cues [12,13]. The classically activated M1 phenotype is known for the release of pro-inflammatory cytokines (Interleukin (IL)-6, IL-1, Tumor Necrosis Factor (TNF)-α), nitric oxide synthase (NOS) and reactive species of oxygen (ROS), etc. [14]. Pro-inflammatory cytokines are able to hinder new bone formation through the inhibition of the osteoblast activity whilst enhancing osteoclast differentiation [15], leading to a delay in the osseointegration process and, over time, implant failure. On the other hand, the alternatively activated M2 phenotype has the opposite effect, enhancing the formation of new bone tissue by downregulating the inflammatory response [11]. Cell debris, byproducts generated during tissue injury and exposure to cytokines, such as IL-4 and IL-13 secreted mainly by mast cells, basophils, and Th2 cells [16], induce a switch in the macrophage phenotype, polarizing the classically activated M1 macrophages into the alternatively M2 macrophages. The M2 macrophages are characterized by high levels of scavenger, mannose and galactose receptors (CD163, CD206, CCR2) [17], high levels in the iron export [18], production of anti-inflammatory cytokines and chemokines such as IL-4, IL-10, IL-13, CXCR1, CXCR2 and expression of arginase-1 (Arg-1), chitotriosidase, mammalian chitinase Ym1, Transforming growth factor (TGF)-β and IL-1 receptor antagonist (IL-1Ra) [19,20,21]. In nature, the M2 macrophage population is heterogeneous, therefore, based on their involvement in wound healing and tissue remodeling, it can be divided into different subtypes (M2a, M2b, M2c, and M2d) [22], each with their own inducers, markers and functions [12,23,24]. The M2a subset (induced by IL-13 and IL-4), secrets high levels of Arg-1 which contributes to the wound healing process through the production of collagen and fibroblast stimulating factors [25]. The second subtype, M2b, is induced by Arg-1, Tool-like receptor ligand (TLRL), and immunocomplexes and is involved in immune response suppression through the production of IL-10 [26,27,28]. Together, the M2a and M2b subtypes exhibit the activation of the Th2 immune response [29]. The M2c subset is induced by IL-10 and glucocorticoids and is involved in the late resolving phase by suppressing the inflammatory reaction through the TGF-β and IL-10 secretion [28]. Altogether, the M2 macrophages subtypes contributes to a suppression in the pro-inflammatory cytokine production, a clearance of cell/tissue debris and a restoration of tissue homeostasis [30]. Moreover, the M2d macrophage subset, also known as tumor associated macrophages (TAM), is capable of secreting the vascular endothelial growth factor (VEGF) and matrix metalloproteinase (MMP), molecules essential in new blood vessel formation and tissue remodelling during the bone repair process [30]. In terms of implantable devices, the presence of the anti-inflammatory mediators and the response following tissue remodeling, aids the vascularization process of the biomaterial through the suppression of the fibrous encapsulation [31]. Therefore, due to their involvement in the immune response modulation, the M2 macrophage prove their important role in tissue repair and bone remodelling process. On that ground, a favourable macrophage phenotype switch can lead to an effective bone tissue regeneration.

Recently, the modulation of macrophage polarization by means of tuning the biomaterial properties has gained special attention in the field of regenerative medicine. The traditional design of biodegradable bone implants focuses mainly on the regulation of bone cells, neglecting the role of the immune system in important processes such as osteogenesis and osteoclastogenesis. Therefore, the immunomodulatory properties of a biomaterial should be taken into consideration when designing new bone implants. Biomaterials with favourable immunomodulatory properties can improve the osteogenic process by inducing an appropriate immune reaction, while biomaterials with poor immunomodulatory properties are able of inducing an inappropriate immune reaction. This last, ultimately may lead to chronic inflammation and implant failure [32] due to the formation of a fibrous capsule around the implant, which restricts the interaction between the implant and the surrounding tissue, therefore making it impossible for the bone cells to attach themselves to the surface of the implant and form new bone tissue. By providing biomaterials with favourable immunomodulatory properties, an improvement in the osteogenic process may be achieved through a favourable immune response. In the last few years, a series of studies focused on the interplay between bone biomaterials and immune cells, trying to evaluate the influence of the macrophages on bone biomaterial-stimulated osteogenesis. However, studies regarding the in vitro osteoimmunomodulatory effects of the bare and coated Mg based biomaterials are fairly limited. In vitro studies on different coated Mg based biomaterials [33,34,35] reported an effectively switch in the macrophage polarization state towards a pro-healing M2 phenotype. Furthermore, the current in vivo studies do not focus on the effect of the bare and coated Mg based biomaterials on macrophages polarization, being confined to the formation of the fibrous capsule and appearance of the inflammatory cells at the implantation site [36,37,38]. Additionally, it is becoming apparent that the in vitro methods used to evaluate the bone implant materials based on their interaction with bone-derived cells are unsatisfactory, as they do not reflect the in vivo conditions which naturally involves an early immune response.

In this study, electrospun poly(ε-caprolactone) (PCL) fibers were functionalized with coumarin (CM) and zinc oxide nanoparticles (ZnO) and deposited on the commercial AZ31 (Mg-3Al-1Zn) Mg-based alloy in an effort to manipulate their immunomodulatory properties. The electrospinning method allows the production of fibers with high porosity, large specific area, and good pore interconnectivity, from polymer solutions or suspensions [39,40,41,42]. These properties, like those of the natural extracellular matrix (ECM), endows the electrospun nanofibers with favourable cell attachment and proliferation abilities [43,44,45]. Electrospun PCL nanofibers have been extensively used in various studies over the last few years, demonstrating their potential as biomaterials for bone tissue regeneration [46,47,48]. Functionalizing the implant surface with bioactive molecules can further improve the osseointegration process. Coumarin is a natural oxygen-containing bioactive molecule, which based on the replacement of different substitutions on the benzopyrone nucleus, exhibits different biological activities [49]. Over the last 50 years, various studies reported the influence of CM on the immune response, cell growth and cell differentiation [50]. Likewise, zinc oxide nanoparticles (ZnO) loaded into electrospun PCL-based membranes have been shown to possess antibacterial activity and a good degree of biocompatibility to human dental pulp stem cells [51]. In this context, the aim of the present study was to investigate the influence of the immune microenvironment generated by the interaction between macrophages and the bare and coated Mg-based biomaterials in terms of cell viability/proliferation and morphology, cytokine protein expression, nitric oxide (NO) and reactive oxygen species (ROS) release. Furthermore, the influence of the immune microenvironment on the macrophage-osteoclast differentiation was investigated by quantification of tartrate-resistant acid phosphatase (TRAP) protein expression and activity, as well as actin cytoskeleton staining of the receptor activator of nuclear factor kappa-B ligand (RANLK)-stimulated RAW 264.7 cells.

## 2. Results

### 2.1. Materials Surface Characterization

**Figure 1:**
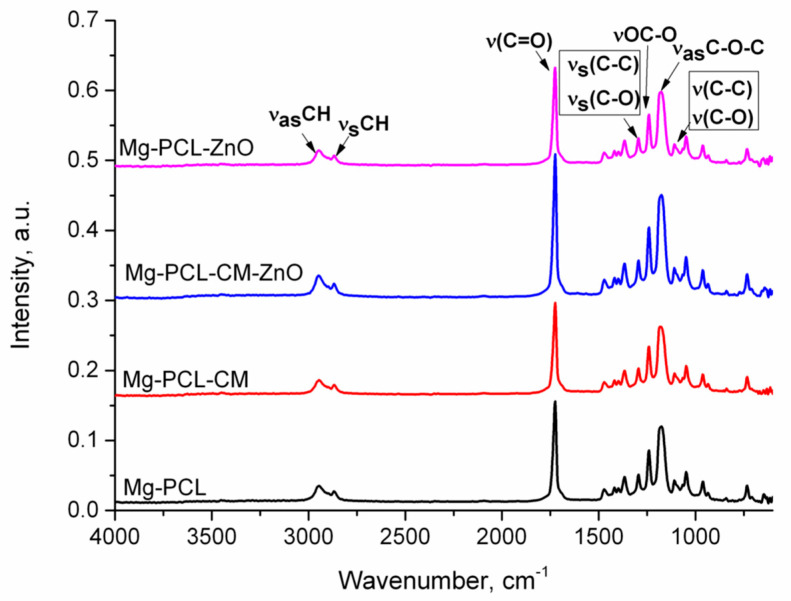
FTIR spectra of Mg-PCL, Mg-PCL-CM, Mg-PCL-CM-ZnO and Mg-PCL-ZnO samples.

Figure 1 shows the FTIR-ATR spectra for Mg-PCL, Mg-PCL-CM, Mg-PCL-CM-ZnO, and Mg-PCL-ZnO samples. FTIT-ATR technique is used to point out the presence of different functional groups which can be found in PCL, CM or ZnO, components that were used to coat the Mg alloy. Due to the low concentrations of CM (5.32 mg mL^−1^) and ZnO nanoparticle (0.25 mg mL^−1^) loaded in the coatings, the corresponding spectra were identical, with PCL’s typical peaks registered at 2945 cm^−1^ and 2868 cm^−1^ (assigned to asymmetric and symmetric –C-H bond from CH2 stretching bands), 1725 cm^−1^ (typical for polyesters and corresponds to the carbonyl ester group), 1294 cm^−1^ (attributed to C-C backbone and C-O stretching band from the crystalline phase), and 1240 cm^−1^ (assigned to C-O-C asymmetric stretching band), 1192 cm^−1^ (characteristic to OC-O stretching vibration), 1168 cm^−1^ (assign to symmetric COC stretching vibration, and 1155 cm^−1^ (attributed to C-O and C-C stretching vibration in the amorphous phase). Another fact of the lack of CM peaks in FTIR spectra is probably the overlapping of its characteristic peaks with the ones of PCL. The characteristics peaks of CM should be present at: 2963cm^−1^ (stretching OH-vibration), 1717 cm^−1^ (C = C stretching vibration), and 1254 cm^−1^ (C-O-C stretching vibration) [52]. The FTIR spectrum of the bare Mg alloy could not be registered.

The presence of CM and ZnO in the PCL coatings was pointed out by X-ray photoelectron spectroscopy (XPS). XPS is a quantitative and qualitative technique that determines the elemental composition of the materials’ surface and also being able to determine the binding states of the elements. Each element (C, O, Zn, Mg, Al) generates a specific group of XPS peaks at a proper binding energy value. These specific peaks are attributed to the electron configuration of the electrons within the atoms, e.g., C 1s, O 1s, Zn 2p, Mg 1s, Al 1p. Therefore, the XPS technique was used to highlight the homogenous surface coverage of the Mg alloy with PCL loaded with CM and ZnO. In order to establish the homogeneity of the sample surface, the analysis was performed in three different points on the same sample, each point having a diameter of 400 μm. Table 1 shows the atomic surface composition of the bare and coated Mg alloy. Mg presents typical atomic elements in their hybridization state, in its composition such as O 1s, C 1s, Mg 1s, and Al 2p, while the coated samples presented only O 1s and C 1s elements. XPS is a surface characterization method that can go in depth up to only 50–70 Å. Therefore, in the case of the coated samples, we analyzed just the fibers that were deposited on the alloy surface. Table 1 presents the average of the atomic percentage from the three analyzed points, of each element that was identified on each sample surface. Taking into account that in coated samples (Mg-PCL, Mg-PCL-CM, MG-PCL-CM-ZnO and Mg-PCL-ZnO) were identified only O 1s and C 1s elements, specific to the polymer (PCL), active ingredient (CM) and ZnO nanoparticle (only O 1s element) and Mg 1s and Al 2p elements were not identified for this samples we may conclude that the alloy surface was perfectly coated with electrospun fibers up to a depth of at least 50–70 Å. The presence of CM was pointed out by the increase of C 1s at.% from 78 at.% (Mg-PCL) to 79 at.% (Mg-PCL-CM) or its decrease to 76 at.% (Mg-PCL-CM-ZnO). The presence of ZnO into the coatings was demonstrated by an increase of O 1s at.% from 22 at.% (Mg-PCL) to 24 at.% (Mg-PCL-CM-ZnO) and 23 at.% (Mg-PCL-ZnO). The small differences in O1s at% and C1s at% between the tested samples can be explained by the small amounts of CM and ZnO loaded into the coatings.

### 2.2. In Vitro Release of Coumarin

The release of CM from the coated samples (Mg-PCL-CM and Mg-PCL-CM-ZnO) was monitored for 24 h in phosphate-buffered saline (PBS). The results showed that the Mg-PCL-CM sample released 15 µg mL^−1^ as compared with 26 µg mL^−1^ released from the Mg-PCL-CM-ZnO sample, which highlights the possibility of tailoring the CM release. The release of a higher amount of CM from the Mg-PCL-CM-ZnO coated alloy took place due to ZnO nanoparticles’ ability to induce defects in the PCL fibers. These defects act like active releasing sites, allowing a higher permeability of the coating to the surrounding PBS, leading to a quick diffusion of the CM molecules.

### 2.3. Electrochemical Behaviour

**Figure 2:**
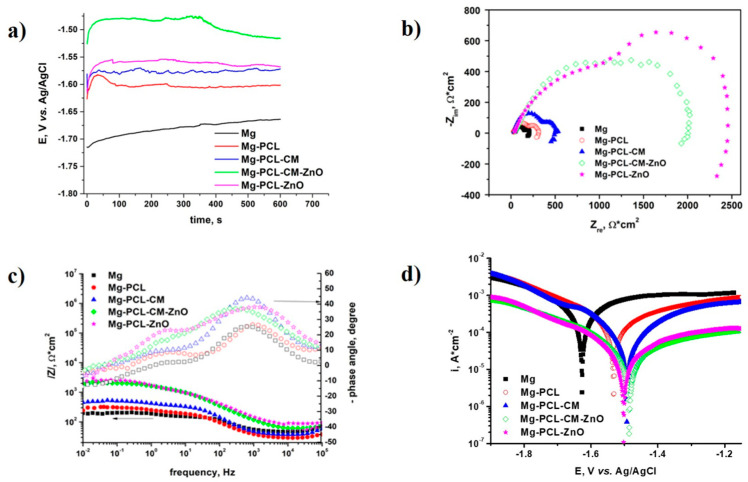
The electrochemical behaviour of the bare and coated Mg alloy in PBS: (**a**) The OCP variation; (**b**) Nyquist diagram at OCP; (**c**) Bode diagram at OCP; (**d**) The polarisation curves.

The open circuit potential (OCP) measurements for the bare and coated Mg alloy in a PBS solution were performed for 600 s. The results obtained are presented in Figure 2a. The electrochemical impedance spectroscopy tests at the OCP in PBS, for the bare and coated samples, led to the recording of the spectra presented in the Nyquist and Bode diagrams in Figure 2b,c. The Nyquist diagrams (Figure 2b) show in all cases, the presence of two capacitive semicircles, one at high frequencies, the other at medium and low frequencies. These two semicircles correspond to the two formed interfaces: one between alloy and coating, and the second between coating and solution. Once introduced into the corrosive environment, the uncoated alloy forms on its surface a magnesium hydroxide layer. This coating being porous and not completely uniform cannot sufficiently protect the Mg alloy against corrosion. In addition, the presence of chloride ions in the electrolyte causes cracks in the hydroxide layer that was formed [53]. The diameter of both semicircles increased in the case of the polymer coated samples in the following order: Mg < Mg-PCL < Mg-PCL-CM < Mg-PCL-CM-ZnO < Mg-PCL-ZnO. The Bode diagram (Figure 2c) revealed the presence of two-time constants corresponding to the two semicircles from the Nyquist diagrams. The first value of the maximum phase angle was recorded at high frequencies, while the second maximum of the phase angle was obtained at lower frequencies. The increase from −25° to −45° of the maximum phase angle value indicated an almost capacitive response of the interface, in the case of the samples covered with polymers (Figure 2c). The polarization curves (Figure 2d) were recorded in PBS for both bare and coated alloy. The anodic oxidation of the Mg alloy can be observed on the anodic branch of the polarization curves. The results show a significant decrease in the value of the corrosion density for the polymer coated samples. Also, a shift in the corrosion potential to more electropositive values in case of the coated samples could be observed.

The kinetic parameters were calculated from the polarization curves (Figure 2d), using two methods: the Tafel slope method and the polarisation resistance method. The values obtained for the corrosion potential (E_corr_), the density of the corrosion current (i_corr_), the penetration index (P), and the polarization resistance (R_P_) are presented in Table 2. The results showed similar values for the corrosion current density, regarding the method used for the analysis. Statistical analysis was performed for all parameters and they are presented as mean ± 1 standard deviation.

### 2.4. Macrophage Viability and Proliferation

**Figure 3:**
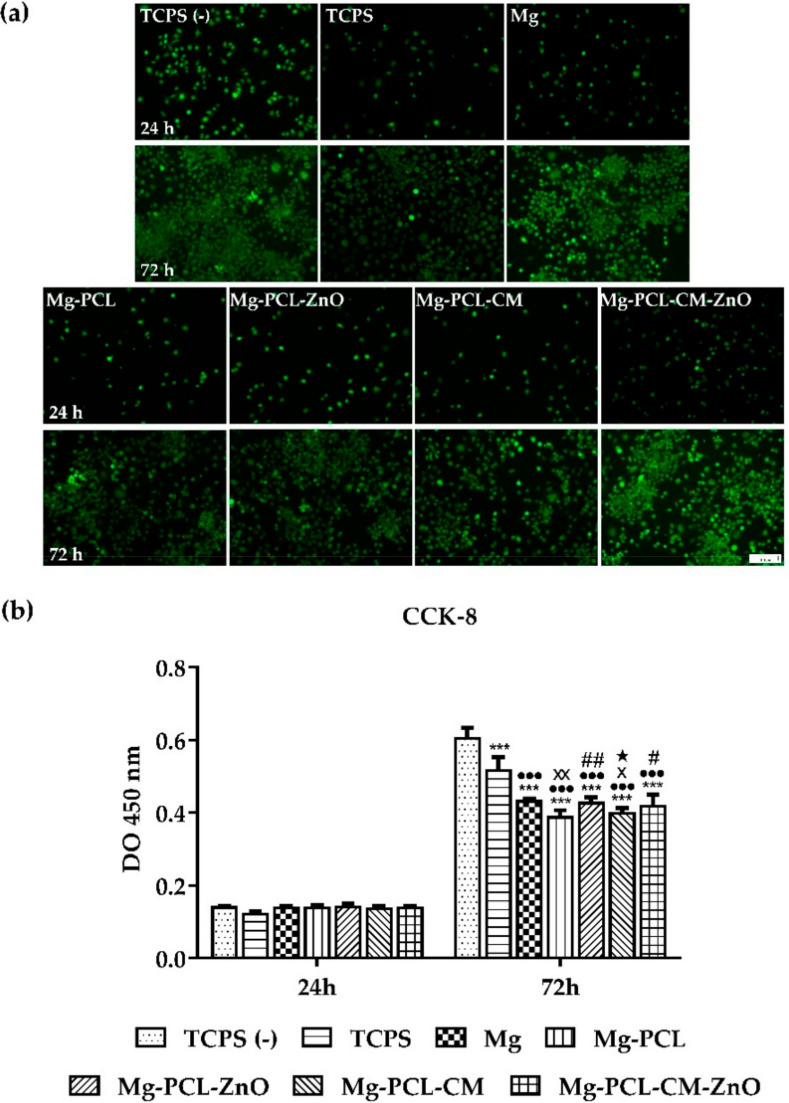
Cell viability/proliferation of RAW 264.7 macrophages grown in culture media containing the extracts of the bare and coated Mg-based alloy, under treatment with LPS (100 ng mL^−1^), and in standard culture conditions (TCPS (−)). (**a**) LIVE/DEAD assay at 1-and 3-days post-seeding (live cells—green fluorescence; dead cells—red fluorescence). Scale bar represents 100 µm; (**b**) CCK-8 assay showing a time-dependent increase in cell proliferation rate without significant differences between the analysed samples at 24 h post-seeding but with significant lower levels in case of the coated/bare samples as compared to those of the control groups under standard (TCPS (−)) and pro-inflammatory (TCPS) conditions after 72 h of culture. The TCPS (−) and TCPS notations denote the negative and positive controls for inflammation, respectively. Results are expressed as means ± SD (*n* = 3, *** *p* < 0.001 vs. TCPS (−); ^•••^ *p* < 0.001 vs. TCPS; ^xx^ *p* < 0.01, ^x^ *p* < 0.05 vs. Mg; ^##^ *p* < 0.01, ^#^ *p* < 0.05 vs. Mg-PCL; ^★^ *p* < 0.05 vs. Mg-PLC-ZnO).

It is well known that the inflammatory response of the adherent macrophages may be influenced by the biomaterial’s ability to support the cellular viability and proliferation. Therefore, to assess the potential cytotoxic effects of the extraction media, the viability of the RAW 264.7 cells was compared by combining calcein AM (acetoxymethyl ester)/EthD-1 (ethidium homodimer-1) staining method (Figure 3a) with the CCK-8 assay (Figure 3b). The results of the LIVE/DEAD assay showed that the extraction media of both bare and coated Mg-based alloy could sustain the survival of RAW 264.7 cells throughout the experimental period, with no observation of red-labelled dead cells. Moreover, a significant increase in the number of cells could be observed over time (Figure 3a). Consistent with the microscopic results, the CCK-8 assay revealed a time-dependent increase in the number of metabolically active viable cells. As shown in Figure 3b, after one day of culture, no statistically significant differences between the tested groups could be observed (*p* > 0.05). However, at 3 days post-seeding, the number of viable, metabolically active macrophages grown in the lipopolysaccharide (LPS) presence both in standard culture medium (TCPS) or in the extracts of the bare and coated samples recorded a significant decrease as compared to the negative control for inflammation ((TCPS (−)) (*p* < 0.001) due to the LPS inhibitory effects on cell proliferation [54]. In addition, significantly less cells were found out in the extracts of the Mg-PCL and Mg-PCL-CM samples than in the extracts of the bare alloy (*p* < 0.01 and *p* < 0.05, respectively). On the contrary, the PCL-ZnO and PCL-CM-ZnO covered Mg alloy exhibited a similar number of viable cells as the bare alloy.

### 2.5. Cell Morphology

**Figure 4:**
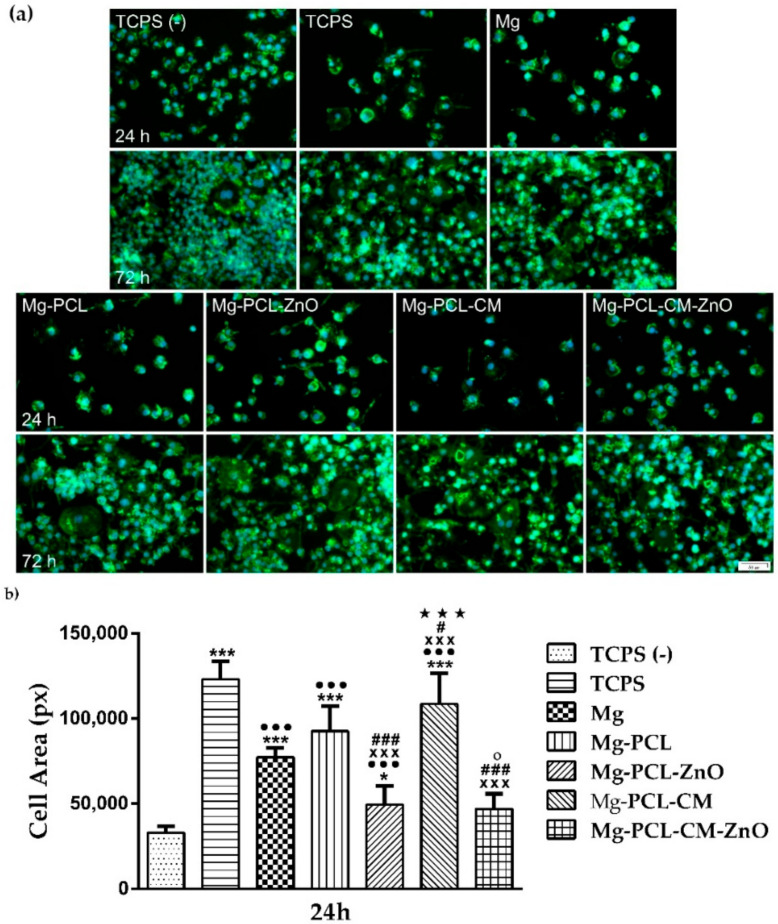
(**a**) Fluorescence images of RAW 264.7 macrophages grown for 24 h and 72 h in standard culture medium in the absence (TCPS (−)) and presence (TCPS) of LPS, as well as in the extraction media of the bare and coated Mg alloy, under treatment with LPS (100 ng mL^−1^). The cells were stained to detect actin cytoskeleton (green) and nuclei (blue). The TCPS (−) and TCPS notations denote the negative and positive controls for inflammation, respectively. Scale bar represents 50 µm; (**b**) Quantification of cell spread area. Results are expressed as means ± SD (*n* = 3; *** *p* < 0.001, * p < 0.05 vs. TCPS (−); ^•••^ *p* < 0.001 vs. TCPS; ^xxx^ *p* < 0.001 vs. Mg; ^###^ *p* < 0.001, ^#^ *p* < 0.05 vs. Mg-PCL; ^★★★^ *p* < 0.001 vs. Mg-PLC-ZnO; ° *p* < 0.05 vs. Mg-PCL-CM).

To evaluate the morphology of the macrophages grown in the analysed extraction media, a fluorescence staining of the actin cytoskeleton was performed after 24 h and 72 h of culture, followed by the visualization under a fluorescence microscope (Figure 4a). In the absence of LPS (TCPS (−)), the cells presented a small and round morphology typical to unstimulated macrophages, while when stimulated with LPS (TCPS), the cells displayed a higher degree of spreading and suffered morphological changes. This enlarged round cell shape with discrete cytoplasmic extensions that can be associated with an activated pro-inflammatory phenotype (M1) were also well-represented in the extraction media obtained from the PCL-CM- and PCL-coated samples and the bare alloy (Figure 4a,b). The same trend was remarked at 72 h post-seeding (Figure 4a). Noteworthy, no significant differences in cell areas were obvious between the samples containing ZnO and the negative control for inflammation (Figure 4b). In terms of distribution, the microscopic observations were consistent with the results of the CCK-8 assay, showing a slight decrease in cell density for the Mg-PCL and Mg-PCL-CM samples.

### 2.6. Multinuclear Foreign Body Giant Cell Formation

**Figure 5:**
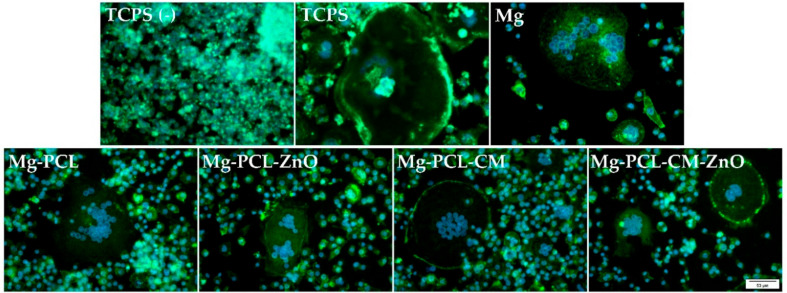
Fluorescence images showing the formation of multinucleated FBGC in standard culture medium (TCPS) and in the extraction media of the bare and coated Mg alloy containing LPS (100 ng mL^−1^) except for the TCPS (−) group. Scale bar represents 50 µm; The TCPS (−) and TCPS notations denote the negative and positive controls for inflammation, respectively.

Foreign body giant cells(s) normally form under a pathological condition, this including the implantation of an orthopaedic biomaterial which will be recognized by the host as a foreign body. Therefore, the formation of FBGCs has been investigated in different studies as a hallmark of the chronic inflammation [8,9,55,56,57,58]. We evaluated the formation of these cells after seven days of incubation in the presence of 100 ng mL^−1^ LPS. Upon this treatment, the RAW 264.7 cells acquired a modified morphology, with larger dimensions and multiple nuclei (Figure 5). Furthermore, the percentage of macrophage fusion was investigated by determining the “multinuclear index”. The values obtained increased in the following order: Mg-PCL-ZnO (1.75%) < Mg-PCL-CM-ZnO (3.1%) < TCPS (8.1%) < Mg-PCL (8.9%) < Mg-PCL-CM (10.2%) < Mg (21.3%) (Table 3). These results indicate that the presence of ZnO nanoparticles in the composition of the coatings led to a less severe inflammatory response. It is worth noting that, in the case of the negative control for inflammation (TCPS (−)), the macrophage fusion phenomenon was absent, the cells remaining mononuclear with a typical round shape and cell body size.

### 2.7. The Extracellular Release of the Pro-Inflammatory Mediators

**Figure 6:**
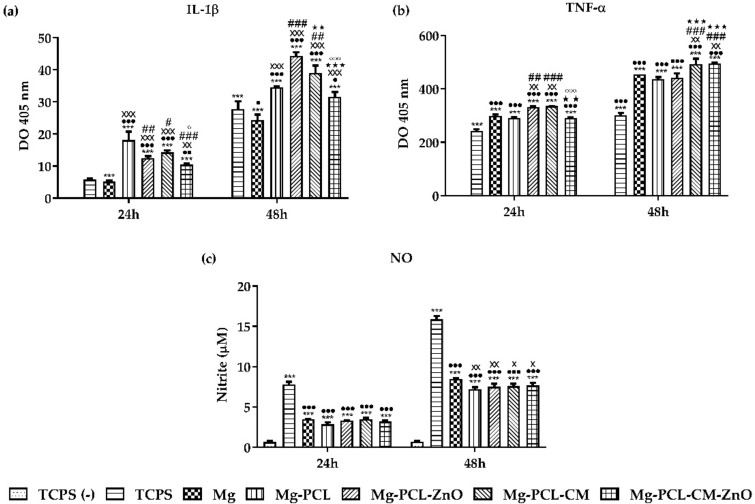
The extracellular release of the pro-inflammatory mediators under treatment with LPS (100 ng mL^−1^) with the exception of the TCPS (−) group: (**a**) IL-1β as assessed by ELISA: *** *p* < 0.001 vs. TCPS (−); ^•••^ *p* < 0.001, ^••^ *p* < 0.01, ^•^ *p* < 0.05 vs. TCPS; ^xxx^ *p* < 0.001, ^xx^ *p* < 0.01 vs. Mg; ^###^ *p* < 0.001, ^##^ *p* < 0.01, ^#^ *p* < 0.05 vs. Mg-PCL; ^★★★^ *p* < 0.001, ^★★^ *p* < 0.01 vs. Mg-PLC-ZnO; ^°°°^ *p* < 0.001, ^°^ *p* < 0.05 vs. Mg-PCL-CM; (**b**) TNF-α as assessed by ELISA procedure: *** *p* < 0.001 vs. TCPS (−); ^•••^ *p* < 0.001 vs. TCPS; ^xx^ *p* < 0.01 vs. Mg; ^###^ *p* < 0.001, ^##^ *p* < 0.01 vs. Mg-PCL; ^★★★^ *p* < 0.001, ^★★^ *p* < 0.01 vs. Mg-PLC-ZnO; ^°°°^ *p* < 0.001 vs. Mg-PCL-CM; (**c**) NO as quantified by Griess diazotization reaction: *** *p* < 0.001 vs. TCPS (−); ^•••^ *p* < 0.001 vs. TCPS, ^xx^ *p* < 0.01, ^x^ *p* < 0.05 vs. Mg. Results are expressed as means ± SD (*n* = 3). The TCPS (−) and TCPS notation denotes the negative and positive controls for inflammation, respectively.

Considering that a certain degree of acute inflammation is required for normal bone healing the cytokine secretion profiles under LPS stimulation were assessed at 1- and 2-days post-seeding by performing an ELISA procedure. As shown in Figure 6, the levels of IL-1β and TNF-α in cell culture media recorded a time dependent increase over the incubation period of two days suggesting an early inflammatory response. In the case of the IL-1β expression (Figure 6a), an approximately two-fold increased level of cytokine production could be seen after 48 h in culture. Furthermore, after 24 h and 48 h of cell incubation, significant differences in the secreted amounts of IL-1β between the analysed cell supernatants could be observed, with the lowest levels recorded in the extraction media from the positive control (TCPS) and bare alloy and the highest levels in the Mg-PCL-ZnO extraction media. Like IL-1β, an upward time dependent trend in the TNF-α expression levels was also recorded over the two-day incubation period. As shown in Figure 6b, significant differences between the analysed samples could be observed at 24 h and 48 h post-seeding. Thus, when compared to the positive control group (TCPS), the tested samples’ extracts recorded significantly increased levels of TNF-α (*p* < 0.001). Additionally, in the absence of LPS in the negative control group (TCPS (−)), the expression levels of IL-1β, and TNF-α were below the assay detection limit, at both experimental time points.

The effects of the extraction culture media on the LPS-induced NO production were evaluated by dosing the concentrations of nitrite accumulated in the culture media, after one and two days of culture. As shown in Figure 6c, the extraction media of all analysed samples induced a significant decrease in NO production when compared to the positive control for inflammation (*p* < 0.001) at both incubation times. In contrast, the control cells grown in the absence of the pro-inflammatory agent (TCPS (−)) showed significantly lower nitrite concentrations in comparison to all the tested samples (*p* < 0.001). Additionally, after 48 h of cell incubation, the extracts of the coated samples induced significantly lower nitrite concentrations in comparison to the bare Mg alloy.

### 2.8. Intracellular Reactive Oxygen Species Generation

**Figure 7:**
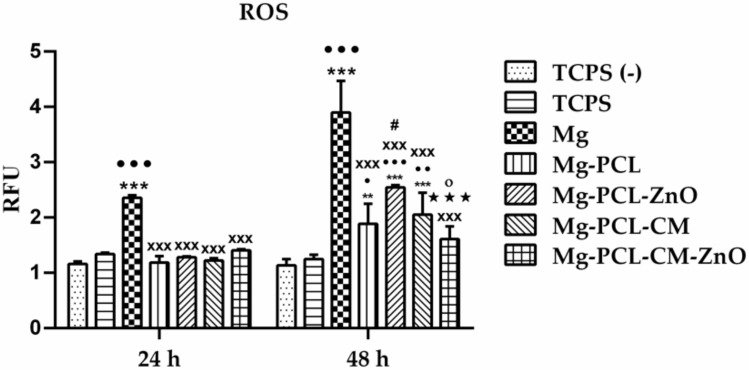
The intracellular ROS levels. Quantity analysis of fluorescence intensity after the DCFDA staining of macrophages stimulated with 100 ng mL^−1^ LPS and exposed to extraction media obtained from bare and coated AZ31 Mg alloy. Results are presented as means ± SD (*n* = 3, *** *p* < 0.001, ** *p* < 0.01 vs. TCPS (−); ^•••^ *p* < 0.001, ^••^ *p* < 0.01, ^•^ *p* < 0.05 vs. TCPS; ^xxx^ *p* < 0.001 vs. Mg; ^#^ *p* < 0.05 vs. Mg-PCL; ^★★★^ *p* < 0.001 vs. Mg-PCL-ZnO; ^°^ *p* < 0.05 vs. Mg-PCL-CM). The TCPS (−) and TCPS notations denote the negative and positive controls for inflammation, respectively.

The levels of intracellular ROS were measured using the fluorescence intensity of dichlorofluorescein (DCF) in order to assess if these Mg-based biomaterials possess the potential to induce oxidative stress in RAW 264.7 macrophages. The results illustrated in Figure 7 show that the bare Mg alloy induced a significant increase of ROS production in a time-dependent manner. So, after the first 24 h of cell incubation, a ~2-fold increased intracellular ROS level was recorded, whereas, after 48 h of exposure, this increase was about three-fold higher as compared to the control samples. To note that after 24 h of culture the extracts of all analysed coated samples induced ROS generation levels like both controls used in the study. On the contrary, after 48 h of culture, these materials excepting the Mg-PCL-CM-ZnO sample, generated significantly higher levels of ROS than the controls. Likewise, no significant differences between the coated samples were noticed excepting Mg-PCL-ZnO versus Mg-PCL and Mg-PCL-CM-ZnO versus Mg-PCL-CM. Furthermore, no significant differences between the macrophages grown in the absence (TCPS (−)) and presence (TCPS) of LPS were observed at both incubation periods.

### 2.9. Macrophage-Osteoclast Differentiation

**Figure 8:**
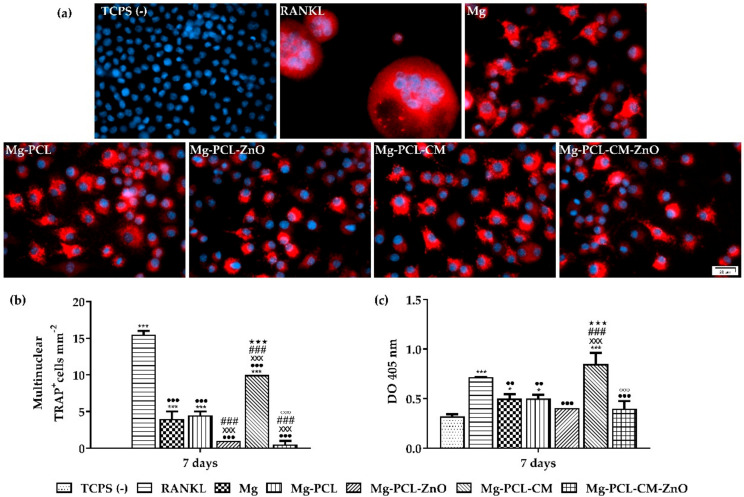
(**a**) Immunofluorescence staining of TRAP (red fluorescence: TRAP signals, blue fluorescence: cell nuclei). Scale bar represents 50 µm; (**b**) The average number of TRAP positive cell per mm^2^. Results are expressed as means ± SD (*n* = 3, *** *p* < 0.001 vs. TCPS; ^•••^ *p* < 0.001 vs. RANKL; ^xxx^ *p* < 0.001 vs. Mg; ^###^ *p* < 0.001 vs. Mg-PCL; ^★★★^ *p* < 0.001 vs. Mg-PLC-ZnO; ^°°°^ *p* < 0.001 vs. Mg-PCL-CM); (**c**) Intracellular TRAP activity. Results are expressed as means ± SD (*n* = 3, *** *p* < 0.001, * *p* < 0.05 vs. TCPS; ^•••^ *p* < 0.001, ^••^ *p* < 0.01 vs. RANKL; ^xxx^ *p* < 0.001 vs. Mg; ^###^ *p* < 0.001 vs. Mg-PCL; ^★★★^ *p* < 0.001 vs. Mg-PLC-ZnO; ^°°°^ *p* < 0.001 vs. Mg-PCL-CM). The cells were stimulated with 50 ng mL^−1^ RANKL, except for the TCPS (−) control sample. The TCPS (−) and RANKL notations denote the negative and positive controls for osteoclast differentiation, respectively.

As osteoclasts serve a vital role in bone metabolism and turnover, being the cells responsible for bone resorption, the effect of the immune microenvironment on the osteoclastogenic process was evaluated by the assessment of TRAP protein expression using immunofluorescence staining at day 7, when RANKL stimulated macrophages were expected to exhibit the characteristics of fully differentiated osteoclasts. Figure 8a shows clear differences between the tested materials. The RANKL positive control media led to the formation of larger TRAP-positive multinucleated cells, while the analysed extraction media, to a different extent, led to the formation of smaller TRAP-positive cells. Furthermore, the count of TRAP-positive cells revealed that the number of osteoclasts increased in the following order: TCPS (−) < Mg-PCL-CM-ZnO < Mg-PCL-ZnO < Mg < Mg-PCL < Mg-PCL-CM < RANKL (Figure 8b). Likewise, the intracellular TRAP activity has also been determined. As shown in Figure 8c, cells incubated in the extraction media obtained from the PCL-ZnO and PCL-CM-ZnO coated alloy presented similar levels of TRAP enzymatic activity to that of cells from the negative control group (TCPS (−)). This finding can suggest that the presence of ZnO in the PCL-based coatings diminished the osteoclastogenesis process. Noteworthy, between the coated samples, TRAP activity expressed by the cells grown in the extraction media from the Mg-PCL-CM biomaterial was significantly higher (~40%) than that exhibited by the rest of the tested samples. Overall, the trend of the TRAP activity is consistent with the trend displayed by the number of TRAP-positive cells.

**Figure 9:**
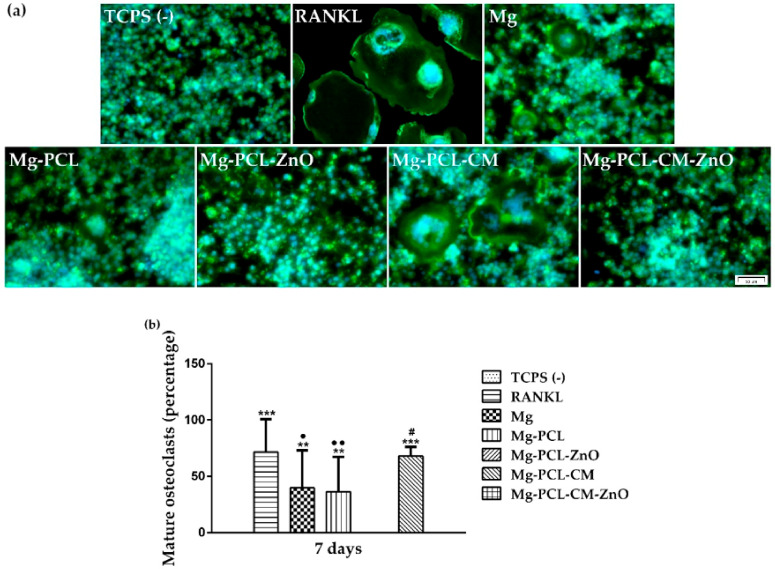
(**a**) Immunofluorescence images of osteoclast formation after 7 days of culture in the presence of RANKL, except for the TCPS (−) sample (green fluorescence: actin cytoskeleton, blue fluorescence: cell nuclei). Scale bar represents 50 µm. (**b**) Quantification of the number of mature osteoblasts. Results are expressed as means ± SD (*n* = 3; *** *p* < 0.001 vs. TCPS; ^••^ *p* < 0.01, ^•^ *p <* 0.05 vs. RANKL; ^#^ *p* < 0.001 vs. Mg-PCL). The TCPS (−) and RANKL notations denote the negative and positive controls for osteoclast differentiation, respectively.

Unlike other cells, mature osteoclasts do not organise their actin cytoskeleton into stress fibers, but instead they form actin rings or podosome belts, structures through which they adhere to the surface of the bone during the remodelling process [59]. These structures can be easily observed after immunostaining with phalloidin. Therefore, the actin cytoskeleton organization was highlighted by fluorescence labelling. The microscopic examination showed significant differences in the size of the actin structures formed in the analysed culture/extraction media (Figure 9a). The RANKL positive control media induced the formation of larger actin rings, while the osteoclasts grown in the extraction media obtained from the bare AZ31 alloy, PCL and PCL-CM coated samples developed smaller actin rings (Figure 9b). Even though these extraction media caused a reduction in the size of the osteoclasts when compared to the positive control group, they did not inhibit the formation of the actin rings. The extraction media obtained from the PCL-ZnO and PCL-CM-ZnO coated samples induced lower effects on the formation of osteoclasts than the bare, PCL- and PCL-CM- coated samples. Moreover, a high number of cells exhibiting morphological features similar to those cultured in the negative control group (TCPS (−)) could be observed in these extraction media, observation being also supported by the results from Figure 9b showing no mature osteoclasts exhibiting actin ring.

## 3. Discussion

Over the last few decades, Mg-based biodegradable metallic implants have received great interest in the field of regenerative medicine due to their bone like properties [60]. However, despite their good mechanical properties and favourable biocompatibility, their use in biomedical applications is limited due to the rapid and non-uniform corrosion in physiological environments [61]. To improve the performance of the biomaterials, surface modifications have been applied with the purpose of attaining a better osteogenic inducing ability. However, osseointegration is a more complex process that requires the help of multiple cells from diverse systems, including immune cells like monocytes/macrophages [62]. As mentioned before, in the last few years, immunomodulation became an important property that endows the bone-implant materials with the capacity of modulating the immune microenvironment in the detriment of a favourable healing process [63]. The biomaterial’s ability to modulate the macrophage polarization has been the subject of several different studies [60,64,65,66,67,68,69,70,71], and even if the direct involvement of immune cells in osteoclastogenesis has been demonstrated, no consensus has been reached on which phenotype is beneficial for a favourable bone regeneration process. Therefore, the present study investigated the interaction between the newly PCL-based coated AZ31 Mg alloy and macrophages in terms of inflammatory response and osteoclastogenic process.

For the polymer-based coated alloys, PCL has been shown to be a biocompatible material which exhibits excellent physicochemical properties such as biodegradability, structural stability, flexible mechanical performance, and low melting point [72,73]. Like other polymers, PCL has been widely applied as a protective coating for Mg alloys to increase the corrosion resistance, being able to suppress gas evolution by acting like a barrier between the surrounding environment and the surface of the biomaterial [74]. This leads to a reduction in the degradation process and the diffusion rate of -OH from the surface to the local environment [75]. Furthermore, the PCL film does not only protect the biomaterials surfaces but it can also provide the required conditions for cell attachment, growth, proliferation and biomineralization by acting as a scaffold [76,77]. Therefore, due to its tailorable properties and suitability for local drug delivery applications, PCL presents an outstanding potential as a promising candidate for the coating of implantable materials.

For further improvement of the biological performance of the Mg-based biomaterials, various bioactive molecules can be incorporated into the polymeric film. Releasing the molecules in a controlled manner can improve the biomaterial’s osteoinductive capacity, stimulate cell migration and promote cell adhesion and proliferation [78]. In this context, the present study aims to functionalize the fibrous PCL coating on AZ31 alloy with CM and ZnO nanoparticles. In the last years, coumarins have attracted a great interest due to their wide spectrum of pharmacological properties such as its antibacterial, antitumor, antioxidant and anti-inflammatory activity [79,80,81]. A large body of data found in the literature, suggests that CM elicits its anti-inflammatory activity by inhibiting the cyclooxygenase (COX) and lipoxygenase (LOX) pathways of the arachidonic acid metabolism [82,83,84]. Likewise, zinc oxide nanoparticles exhibit more favorable osteogenic properties compared to their bulk counterparts owing to their small size and large specific surface area. Furthermore, they possess anti-inflammatory [85] and pro-angiogenic [86] activities. ZnO nanoparticles assimilated by endocytosis are dissociated releasing Zn^2+^. Zn has been shown to exert inhibitory effects on the osteoclast formation by suppressing the Ca^2+^-Calcineurin-NFATc1 signaling pathway [87].

The FTIR analysis of the tested materials’ surfaces showed similar spectra with all the signals corresponding only to the PCL polymer due to the technique’s inability to identify the loaded CM and ZnO nanoparticles. This phenomenon was also reported by Reyers-Lopez et al. [88] on PCL electrospun nanofibers loaded with gold nanoparticles. The XPS results indicated the perfect coating of Mg alloy with the electrospun PCL mixtures by the presence only of O1s and C1s atoms. The modification of the percentage of these atoms in the case of the coated samples demonstrated the presence of CM and ZnO into the PCL fibers [89].

As it can be seen from OCP behaviour, in the very first few seconds a fast shift of the potential towards more electropositive values was recorded, followed by a slight shift towards more electronegative values, and ending with a stabilized value. Reaching a stable value relatively quickly can be explained by the high dissolution rate of the Mg alloy, meaning a low time constant related with the charging/setting of the electrochemical double layer [90]. Compared to the bare AZ31 alloy, the coated samples recorded more electropositive values of the potential, with the PCL-CM-ZnO coated sample exhibiting the most electropositive stable value. The diameter of the two semicircles obtained in the Nyquist diagram recorded in PBS increased significantly for the coated samples, indicating a higher increase of the polarization resistance, which led to higher decreases in the corrosion rate. The Nyquist diagram showed the presence of an inductive loop recorded at a low frequency for all tested samples because of the pitting corrosion initiation [91]. Furthermore, the corrosion resistance could be estimated from the Bode diagrams. A higher value of the impedance modulus indicated a better anticorrosion property [92]. As seen from the Bode plots, the PCL-ZnO and PCL-CM-ZnO coatings have the highest impedance modulus values at 0.01 Hz, and therefore, the best anticorrosion property in PBS. By comparing the behaviours of the two coatings with the best protection, it can be noticed that, while the maximum phase angle values recorded at the high frequency remain almost constant, the ones recorded at low frequencies increase slightly for the PCL-ZnO coated alloy, indicating better protection against corrosion. This finding reconfirms what can be more easily observed from the Nyquist diagram, showing that the diameter of the semicircle increases for Mg alloy coated with PCL-ZnO. From the polarization curves, it can be observed that for all coated samples there is a shift in the anodic branch by principally retarding anodic dissolution. It can be said that all types of coatings studied can serve as a functional anodic coating. Moreover, the best corrosion behaviour was exhibited by the Mg-PCL-ZnO sample. In this case, the lowest value of the corrosion rate and, correspondingly, the highest value of the polarization resistance were found out. Additionally, the PCL-CM-ZnO coating offered a favourable corrosion behaviour for the AZ31 Mg alloy. The corrosion current density values for the two coated samples are quite close and with an order of magnitude lower than the corrosion current density value recorded for the bare alloy. Moreover, the corrosion rate of the Mg-PCL-ZnO sample, calculated from the Tafel tests, was 0.127 mm/year, much slower than the rate recorded for the bare alloy (1.751 mm/year). These data confirm the results obtained from the electrochemical impedance spectroscopy tests and are like the results obtained by other researchers for other types of polymeric coatings deposited on biodegradable Mg alloys [93,94]. Thus, the PCL-ZnO and PCL-CM-ZnO coated samples exhibited the best corrosion behaviour in a PBS solution.

To predict the in vivo behaviour of the bone implants, in vitro studies are often performed before biomaterial implantation. Witte et al. [95] demonstrated that, in case of Mg-based materials, direct contact assays affect cell viability more rapidly than indirect contact tests (the extraction method). On this ground, in the present study, the in vitro cellular response was investigated using the extraction method, in accordance with the ISO 10993-12 standards [96]. However, to protect the cells against the osmotic shock generated by the corrosion products released into the culture medium and to better reproduce the in vivo conditions, where these products could be tolerated due to circulatory system of the body, a dilution of the extraction media is highly recommended [97]. Moreover, the extraction should take place in cell culture media supplemented with serum, since the use of salt solutions will only increase the corrosion process of the biomaterials [98]. Therefore, in this study, the extraction media were obtained by incubating the samples with cell media supplemented with 80% serum, with a view to create an environment as close as possible to the in vivo situation in terms of albumin and other biomolecules concentrations [98]. To investigate the influence of the bare and PCL-based coated AZ31 Mg alloy on the immune response, the cellular model was represented by the monocyte/macrophage cell line RAW 264.7. In the specialised literature, studies reported the use of the human monocytic cell line THP-1 [99,100,101,102,103]. However, the mouse RAW 264.7 cell line, regardless of its transformed phenotype, is generally used in inflammation studies due to its response to *Escherichia coli* LPS which can imitate bacterial stimuli [98,104,105,106]. Additionally, Berghaus et al. (2010) reported that RAW 264.7 cells are capable of mimicking bone marrow-derived macrophages in terms of cell surface receptors and response to several microbial ligands [107]. The RAW 264.7 cells were grown in culture media containing the diluted extracts, and their behaviour was firstly assessed in terms of cell viability/proliferation, cell morphology, pro-inflammatory cytokines production, as well as ROS and NO production. In these experiments, the macrophages were stimulated with LPS (excepting the negative control for inflammation (TCPS (−)), a molecule of bacterial origin that activates the macrophage M1 phenotype and is generally used in inflammation related studies [14].

The cell viability and proliferation assays showed that the extraction media of the tested samples supported the survival and proliferation of the RAW 264.7 cells, without any significant differences between the tested samples after 24 h of culture. To note that a significant increase in the number of metabolically active viable cells was recorded at 72 h post-seeding and that the control groups showed the highest proliferation potential. Jenkins et al. [108] suggested that a good in situ proliferation is important, especially in the early stages of inflammation when an accumulation of macrophages occurs, contributing to the wound repair process. Fluorescence images of the actin-labelled cells revealed that under LPS-treatment, the RAW 264.7 macrophages acquired the morphological features of the M1-pro-inflamatory phenotype [14,48,109] in the positive control for inflammation and to varying degrees in the extraction media of the analysed Mg-based materials. Thus, this morphological phenotype was dominant in case of the TCPS sample where the cells exhibited the greater cell area and was also remarked in extraction media of the Mg-PLC-CM, Mg-PCL, and Mg materials. The higher degree of cell spreading and larger dimensions could suggest that the combination of these extracts and LPS treatment led to a stronger inflammatory activity of RAW 264.7 cells, compared to the rest of the tested samples. It is important to note that the coatings containing ZnO nanoparticles, namely PCL-ZnO and PCL-CM-ZnO, predominantly showed a small-rounded morphology typical to unstimulated macrophages as noticed in the absence of LPS (TCPS (−)). However, even though the cellular morphology is seen as a characteristic of the inflammatory response towards the biomaterial [81], recent data revealed that a modified cell morphology does not necessarily indicate a strongly activated macrophage phenotype [110].

Additionally, further investigations were conducted to evaluate the biomaterials’ influence on the inflammatory activity of the RAW 264.7 cells. The host immune reactions after materials’ implantation include a series of events such as blood-material interaction, provisional matrix formation, acute and chronic inflammation, formation of the granulation tissue, foreign body reaction and fibrous capsule formation [55,111,112,113,114]. Following in vivo implantation, proteins such as albumin, fibronectin, vitronectin, fibrinogen, complement proteins, and globulins [115,116,117] are adsorbed on the surface of the biomaterial, leading to the formation of a provisional matrix [118] rich in growth factors, cytokines, and chemo-attractants capable of recruiting innate immune cells to the site [8]. In the next step, neutrophils migrate to the implantation site, indicating the beginning of the acute inflammatory response, which usually lasts less than one week [8]. Following this state, a prolonged recruitment of other types of immune cells can lead to a chronic inflammatory response. When the inflammatory state has reached its end, the granulation tissue starts to form, leading in the end to fibrous capsule development and implant failure. One of the dominating infiltrating cells at the implantation site are macrophages [119], capable of responding and binding to almost any kind of implantable biomaterial (e.g., metals, polymers, ceramics, collagen) [120,121,122,123]. However, the mechanisms by which they recognize the implantable devices are still not completely elucidated. One of the hypotheses suggests that following implantation the proteins from the hosts are adsorbed on the surface of the biomaterial [124] and trigger macrophage activation and sequentially their response. A second mechanism proposes the formation of complexes between the macrophages’ complement receptors and adsorbed complement proteins or IgM and IgG antibodies [55], while the last mechanism suggests that the cell adhesion mediated by ligand-receptor complexes is modulated by growth factors and cytokines [125]. The mechanism through which macrophages bind to foreign bodies is known and it involves transmembrane proteins called integrins [118]. These proteins mediate the interaction between cells and the extracellular matrix, and the bond between themselves and the surface of the biomaterial leads to modifications in cell movement, cellular survival and proliferation and changes in the actin cytoskeleton [55]. Adhesive structure and cytoskeleton studies reported that the major adhesive structures involved in the adhesion of macrophages to the surface of the biomaterial are podosomes and not focal contacts [125]. Once adherent to the surface of the biomaterial, the macrophages will try to destroy the foreign body, but unable to engulf the whole biomaterial as single cells, they will fusion and form FBGCs. Similar to the recognizing mechanism, the fusion process has not been fully elucidated, but a hypothesis has been advanced where it has been suggested that the process has three distinctive steps. In the first step the cells acquire the ability to fuse through the DAP12/Syk signalling pathway activation [126], then the cell will migrate and adhere to other cell’s membranes and lastly the cells become one single entity with common cellular components [127] and a reorganized actin cytoskeleton which will permit cell spreading and motility [128]. FBGCs present filamentous actin bundles called filopodia, involved in the chemotactic sensoring, cell motility and surface adhesion [129,130]. At a structural level, the fusion process requires a close contact and disruption of cell membranes, which occurs due to the interaction between calcium ions and soluble NSF attachment protein receptors (SNAREs). The interaction will lead to the formation of conducting channels through which the calcium bridges from apposing bilayers will release water from hydrated Ca^2+^ ions capable of disrupting the cell membrane [131]. Regarding the fusion mechanism, the formed FBGCs can cause chronic inflammation by releasing degradative reactive oxygen intermediates, MMPs and inflammatory cytokines [56] thus leading to an impaired new bone formation and biomaterial degradation [55]. Furthermore, a prolonged inflammatory response causes fibroblast infiltration and collagen synthesis, leading to fibrous capsule development [132]. The formation of the fibrous capsule will prevent the formation of new bone tissue by disrupting the interaction between biomaterial and bone cells. On this ground, we investigated the ability of the analyzed samples to induce the macrophage fusion and form undesirable FBGCs. The results show that in a microenvironment that mimics bacterial contamination by LPS treatment, the extraction media obtained from the samples containing in their coating ZnO nanoparticles led to a lower degree of macrophage fusion suggesting that they do not support the transition from acute inflammation to an exacerbated chronic inflammation resulting the implant failure [8]. This response could be attributed to the anti-bacterial [133] and anti-inflammatory capabilities [134] of the ZnO nanoparticles. Given the known anti-inflammatory activity of ZnO nanoparticles, studies have reported their dose—and size dependent effects on the inflammatory response of RAW 264.7 cells, with lower doses and smaller sized nanoparticles (30 nm) having the most beneficial effect [134,135,136]. Even though the anti-inflammatory activities of different CM compounds have been reported in several studies [50,136,137,138,139] here, the extraction media obtained from the PCL-CM coated sample exhibited a more pronounced pro-inflammatory response close to the TCPS sample but two-fold lower than the bare alloy, a feasible explanation being the lower amount of CM released from the PCL-CM coating (15 µg mL^−1^) in comparison to the PLC-CM-ZnO coating (26 µg mL^−1^).

Immediately after bone trauma, the fracture healing process involves an initial haematoma formation followed closely by the activation of the immune system [140,141,142]. The inflammatory phase appears within the first 12 to 14 h after bone injury, reaching its peak during the first 24 h and it is characterized by a local production of cytokines and growth factors that initiate the healing process of the injured tissue [143]. During this time, an up-regulation of TNF-α, IL-1, IL-6, IL-17, and IL-18 occurs [144,145]. Although, an increased production of pro-inflammatory mediators is generally associated with chronic inflammation and tissue destruction [146,147] their activation during the early inflammatory phase initiates the normal healing process by stimulating the proliferation of the mesenchymal stem cells (MSCs) and their differentiation into mature osteoblasts [142]. Even though the acute inflammatory response plays an important role in the initiation of the fracture healing cascade, after a few days the initial haematoma is cleared and replaced by a granulation tissue rich in proliferating MSCs [148,149]. In this proliferative phase, the macrophages switch their phenotype to M2 macrophages and start to produce anti-inflammatory mediators involved in tissue regeneration [150]. Therefore, an adequate inflammatory response represents a necessary phase for the bone repair process [151,152]. In this context, the short-time secretion of the pro-inflammatory cytokines IL-1β and TNF-α has been investigated under LPS-stimulation. IL-1β promotes the production of the primary cartilaginous callus in the early inflammatory stage [153,154] and influences the production of IL-6 [153]. TNF-α is involved in signalling induction during the secondary inflammatory phase, recruiting cells necessary for bone regeneration [154]. Additionally, coupled with IL-6 production, it promotes in vitro matrix mineralization and in vivo bone regeneration [154,155]. The bimodal role of the pro-inflammatory response contributes to its significance in the bone regeneration process and the inhibition of the early inflammatory process is normally associated with poor healing [156,157]. Our data showed that the IL-1β and TNF-α levels recorded a progressive accumulation in the culture media along the incubation period of 48 h. It is worthy to mention that the secreted levels of cytokines in the extraction media from the majority of coated AZ31 samples were higher than those expressed by the LPS stimulated positive control group (TCPS) suggesting a more inflammatory initial macrophage response. However, taking into consideration the results of the FBGC formation it can be considered that these samples do not sustain a chronic inflammatory response. On the contrary, the bare Mg alloy induced the secretion of almost similar levels of IL-1β and of higher amounts of TNF-α than TCPS sample but at the same time exhibited the highest multinuclear index. Constantino et al. [158] investigated the in vivo effect of various Mg extracts on cytokine production and reported an increased level of IL-1β at 24 and 72 h, thus indicating that Mg based biomaterials are capable of influencing macrophage-cells activity at a molecular level. More recently it has been demonstrated, in a mouse tibial fracture model, that the expression pattern of TNF-α and IL-1β, following bone injury, has two peaks: the first peak emerges within the first 24 h and lasts for almost 72 h, while the second peak occurs after 3–4 weeks [155]. In a similar fracture model, Glass et al. [159] reported that local administration of TNF-α promoted the recruitment and differentiation of muscle-derived stromal cells, while Gerstenfeld et al. [160] demonstrated that TNF-α knockout mice have shown impaired fracture healing. Likewise, Lange et al. [161] demonstrated enhanced proliferation of osteoblasts and bone matrix mineralization coupled with a reduced proliferation and differentiation of MSCs after IL-1β stimulation. We presume that the varying amounts of cytokines secreted in the extracts of the developed Mg-based materials are due to the release of the corrosion byproducts and functionalizing agents. It is generally acknowledged that free zinc (Zn) ions are involved in the production of pro-inflammatory cytokines such as IL-6, IL-1β and TNF-α [162]. Therefore, we postulate that the cytokine profiles of the samples containing ZnO within the PCL coatings could also be attributed to the presence of ZnO nanoparticles and the release of Zn ions from their surface. Hanley et al. [163] investigated the mechanism of cytokines induction by ZnO nanoparticles and their results showed that the induction of immunoregulatory cytokines such as TNF-α is concentration dependent. Likewise, Heng et al. [164] reported that the induction of inflammatory cytokines such as TNF-α by ZnO nanoparticles is dose-, shape-and size-dependent, with the higher concentration (30 µg/mL), spherical shape and average size being capable of enhancing the levels of TNF-α up to 200 times higher than the control group. However, the data available in literature regarding the influence of Zn on the inflammatory cytokine production is highly contradictory [165]. Additionally, Jin et al. [100] reported that high concentrations of degradation products released in the culture media promoted the TNF-α expression, mainly due to the early foreign body response (FBR) process. Moreover, Ralston et al. [166] reported that high levels of IL-1 are associated with low concentrations of NO, therefore justifying our findings regarding the high levels of this mediator induced by the extracts of the developed Mg-based materials, which correspond to lower levels of NO production. NO is a gaseous messenger molecule biosynthesized from L-arginine and molecular oxygen by three different NO synthases (NOS). The NOS isoforms include neuronal nitric oxide synthase (nNOS), endothelial nitric oxide synthase (eNOS), and inducible nitric oxide synthase (iNOS), with iNOS generally expressed under LPS and interferon-γ stimulation [167]. Its involvement in bone turnover is paradoxical, due to the fact that high levels of NO have been associated with bone loss in some inflammatory conditions but NO also elicits beneficial effects on bone via NO/cyclic guanosine monophosphate (cGMP) pathway [168]. Our results showed significant differences in terms of NO release for the cells grown on TCPS under pro-inflammatory (TCPS) and standard (TCPS (−)) conditions where the highest and, respectively, lowest levels of nitrite concentration could be observed both at 24 h and 48 h. Moreover, the bare Mg alloy exhibited a slightly increased level of nitrite released into the media, when compared to the coated samples. The results suggest that by coating the AZ31 Mg alloy surface, the production and accumulation of NO in the culture media could be impeded.

In addition to all these inflammatory mediators, LPS-stimulated macrophages also produce ROS [169]. It has been recently shown that the degradable particles derived from Mg-based alloys can be up-taken by macrophages and induce a higher level of TNF-α in culture conditions [100], generating an inflammatory response and ROS overproduction [170]. Although it is well known that excessive generation of ROS is cytotoxic, it is believed that sub-lethal levels of ROS act as second messengers in signalling cascades involved in genes expression [171]. Furthermore, the generation of ROS on the surface of biomaterials may be involved in creating optimal conditions for the successful osseointegration of different implants [172]. Likewise, a recent study has indicated that ROS overproduction on bare Mg may indirectly control the cell morphology and cell adhesion molecules, while moderate levels of ROS around the implant’ surface are useful for cellular proliferation [173]. In this context, our finding that the extracts of the coated samples induce moderate intracellular ROS levels when compared to the control samples and the bare Mg alloy could support their successful future use as next-generation biomaterials for bone repair and reconstruction. The decreased ROS levels in cells exposed to the extracts obtained from Mg-PLC-CM and Mg-PLC-CM-ZnO samples could be explained by the antioxidant effect of coumarins based on their ROS scavenging activity [174]. On the other hand, increased levels of Zn could diminish ROS presence because it inhibits NADPH-oxidase [175] and stimulates the expression of metallothioneins, which quench ROS by oxidation of cysteine residues [176].

Apart from regulating the immune response, macrophages can control the osteogenesis and osteoclastogenesis processes through the release of a wide range of molecules. Over the last few years, it was discovered that the interaction between the immune system and bone cells in the late stage of the inflammatory process, plays a key factor in the formation of new bone tissue. Consequently, another objective of the present study has been to investigate the influence of the immune microenvironment generated by macrophages incubated in the extraction media obtained from the developed Mg-based biomaterials on the osteoclastogenic process. Like the natural bone remodelling, the successful implantation of an orthopaedic device requires a coordinate activation of bone-forming osteoblasts and bone-resorptive osteoclasts [177]. Therefore, understanding the mechanism through which osteoclasts interact with the implantable biomaterials would be beneficial for developing a suitable implant surface capable of regulating the osteoclastogenic process towards a desirable result [178]. An impaired interaction can tip the balance from osteogenesis towards osteoclastogenesis, thus leading to a delay in fracture healing which can cause post-surgical complications such as mal-union, implant loosening and, finally, implant failure [179,180,181]. The primary cells in the osteoclastogenic process are represented by the osteoclasts, which in comparison to osteoblasts, are less studied and less understood. Osteoclast differentiation and activation are mediated by various cytokines and growth factors. Among them, RANKL, a member of the TNF superfamily, osteoprotegerin (OPG) a decoy receptor, and macrophage-colony stimulating factor (M-CFS) play a key role [59,182,183]. RANKL and OPG form the RANKL/RANK/OPG system, in which the interplay between RANKL/RANK leads to osteoclast differentiation, while OPG binding leads to osteoclast suppression [184]. For many years it was thought that RANKL was secreted only by the osteoblasts, probably due to co-culture studies where formation of mature osteoclasts from bone marrow macrophages was observed [185], but several studies implied that osteocytes were also capable of secreting RANKL and supporting osteoclastogenesis [186,187]. Osteoclasts originate from bone marrow myeloid progenitor cells [188], which under the influence of M-CSF and RANKL undertake an osteoclast phenotype [189]. Following RANKL binding to receptor activator of NF kappa B (RANK), TNF receptor associated factor 6 (TRAF6) is recruited to the RANK intracellular domain, leading to the activation of the nuclear factor kappa B (NF-kB) and mitogen-activated kinases, such as p38 and Jun n-terminal kinase (JNK) [190,191,192,193]. Once activated NF-kB modulates the transcriptional activity of nuclear factor of activated T-cells cytoplasmic 1 (NFATc1), and its inactivation or depletion will result in a failed osteoclast differentiation [194]. Furthermore, NFTAc1 is capable of binding to its own promoter, enhancing the differentiation process with the help of an activator protein 1 complex c-Fos [195]. Under this scenario, in the present study, RAW 264.7 macrophages were seeded and cultured in the corresponding extraction media in the presence of 50 ng mL^−1^ RANKL for seven days and analysed for differentiation in mature osteoclasts expressing TRAP activity and actin ring. For many years TRAP protein has been used as an important marker of osteoclasts due to its secretion during bone resorption [196,197]. Even though TRAP is generally released in the surrounding media, high quantities of protein can still be found in the active osteoclasts. Therefore, the TRAP protein expression was first investigated by means of immunofluorescence staining. The fluorescence images revealed the presence of numerous TRAP-positive cells in the extraction media obtained from the bare, as well as the PCL- and PCL-CM- coated AZ31 alloy. By contrast, the frequency of TRAP-positive cells was significantly reduced in the extraction media obtained from the PCL-ZnO and PCL-CM-ZnO coated alloy. As expected, the RANKL positive control media led to the formation of larger multinucleated cells, suggesting that in the case of the extraction media a reduction of the osteoclast maturation occurred. Consistent with the microscopic results, our data on the quantification of the TRAP-positive multinucleated cells and TRAP activity showed significant differences between the analysed samples, with the Mg-PCL-CM exhibiting the highest levels. This result is in line with data reported by Lee et al. [138] showing that CM promoted the formation of TRAP-positive multinucleated cells. Noteworthy, the co-presence of ZnO nanoparticles in the PCL-CM and PCL-CM coatings induced the most significant decrease in the mentioned two osteoclast-specific markers. Further on, RANKL-induced osteoclast actin ring formation was investigated. The osteoclast actin ring is one of the most visible morphological features of mature osteoclasts and plays an essential role in the bone resorption process [198,199]. The initial interaction between osteoclasts and bone matrix is mediated by distinctive dot-like F-actin rich structures called podosomes [200]. These structures are involved in processes like cell adhesion, extracellular matrix degradation and cell migration [201]. During the remodelling process the osteoclasts polarise and in the intermediated stages, the podosome cluster rearranges to a disk-like pattern called podosome ring, which will become encircled by a single actin ring and a double vinculin ring during the late stages of the differentiation process [177,200,202]. The newly developed rings form a new structure called a podosome belt. Also known as the actin ring, the structure attaches the cells to the surface of the natural bone, isolating the acidified resorptive microenvironment from the extracellular environment during the resorptive process [59,203]. The actin-phalloidin staining revealed smaller sized actin ring structures for the cells grown in the extracts of the bare, and PCL- and PCL-CM- coated AZ31 alloy in comparison to the structures observed in the RANKL positive control media. These results are in good agreement with the results regarding TRAP protein expression and activity and suggest that the developed biomaterials are variably able to suppress the actin ring formation. It is important to note that the extraction media obtained from the Mg-PCL-ZnO and Mg-PCL-CM-ZnO samples did not induce actin ring formation like the negative control for osteoclast differentiation, thereby indicating their capability to suppress the excessive bone resorptive activity. Overall, our findings on the osteoclastogenic potential of the developed fibrous PCL-based coatings on the AZ31 Mg alloy are not surprising, being consistent with the previously reported data. For instance, Zhai et al. [198] demonstrated that pure metallic Mg suppressed osteoclast formation, polarization and bone resorption in vitro, while Wu et al. [204,205] showed that the inhibitory effect on the osteoclast differentiation has a direct correlation to the Mg concentration.

## 4. Materials and Methods

### 4.1. Materials

The commercial AZ31 magnesium alloy (94 wt.% Mg, 3 wt.% Al and 1 wt.% Zn) used in this study was purchased from Sigma Aldrich as a foil of 10 × 10 cm. From sheets of 1 mm thick, samples of 1 × 10 cm were cut for the coating procedure. Each sample was sanded using SiC abrasive paper with an increasing granulation (400–1200 grain), followed by an immersion for 10 min in absolute ethanol for cleaning. Poly(ε-caprolactone) pellets (Mw = 80,000 g/mol), CM, ZnO nanoparticles with an average diameter of 20–30 nm dichloromethane (DCM), acetic acid (Acetic Ac.) and ethanol were purchased from Sigma Aldrich and used as received. For the electrochemical characterisation, PBS (pH 7.4) with the chemical composition presented in Table 4 was used as electrolyte [206].

### 4.2. Coating Precursor’s Preparation

A PCL stock solution of 15 wt.% was prepared by solubilizing the polymer in a DCM:Acetic Ac. (70:30) solvent mixture. Subsequently, the stock solution was mixed with distilled water in a ratio of 19:1 (*v*/*v*). Coumarin solubilization (5.32 mg mL^−1^) and ZnO dispersion (0.25 mg mL^−1^) in the PCL stock solution led to the formation of the biocomponent systems, Mg-PCL-CM and Mg-PCL-ZnO, while the three-component system, Mg-PCL-CM-ZnO, was prepared by adding both CM and ZnO, respectively. The prepared blends are presented in the Table 5.

### 4.3. Electrospinning Fabrication of the PCL-Coatings

A climate-controlled electrospinning equipment (EC-CLI, IME Medical Electrospinning, Waalre, The Netherlands) was used for the deposition of the fibrous coatings on the Mg alloy surface. Before the electrospinning process, to remove the native oxide layer, the surface of the alloy was sanded and washed extensively with ethanol. Following this step, the Mg plates (100 × 10 × 1 mm) were attached to the grounded rotary collector support and the electrospun fibers were obtained by loading approximately 3 mL of each mixture in a 5 mL plastic syringe, placed on the syringe pump in order to precisely control the flow rate of the injected blends. All solutions were injected through a nozzle of 0.4 mm inner diameter, and 500 µL of each blend was electrospun at a constant temperature of 23 °C and a relative humidity of 50%, maintained throughout the entire experimental period. For a homogenous deposition of the fibers, the following non-variable parameters were used for each composition: rotating mandrel of 75 rpm, tip to-collector distance of 12.5 cm, voltage of −4 kV applied on the collector, nozzle speed of 5 mm∙s^−1^ along the entire length of collector. The voltage applied on the nozzle tip of the syringe and the flow rate varied, depending on the injected composition. The established parameters for each composition are shown in Table 6.

### 4.4. Spectrometric Characterization of the Coated Alloys

Attenuated total reflection Fourier-transform infrared (FTIR-ATR) spectra were registered with Bruker VERTEX 70 spectrometer, acquiring 32 scans with a resolution of 4 cm^−1^ in 600–4000 cm^−1^ range. The ATR module is equipped with a reflection Ge crystal. The baseline is made in the same measurements conditions as the tested sample. Before the measurement of each sample, the crystal is cleaned and the bare and coated Mg alloy are placed over the Ge crystal, which has a high index of reflexion that allows the internal reflexion within the sensor. Therefore, when the IR beam arrives at the sample’s level, which has a low refractive index, a part of the light is reflected back.

The XPS analysis were performed on a K-Alpha instrument from Thermo Scientific (USA) with a monochromated A|K_α_ source (1486.6 eV) and working at a vacuum base pressure of 2 × 10^−7^ mbar, equipped with a flood gun to compensate the charging effect. XPS provided information regarding the electron energy distribution in the obtained bare and coated Mg alloy.

### 4.5. In Vitro Release of Coumarin

The in vitro release of biomolecules like CM from the coated alloy samples was monitored after 24 h in PBS. Thus, the coated samples that contained CM (Mg-PCL-CM and Mg-PCL-CM-ZnO) were cut at a dimension of 3 cm^2^ and submersed in 1 mL PBS for 24 h at 37 °C and 75 rpm in a thermostated shaking water bath GFL 1083. Afterwards, the supernatant was extracted, diluted up to 3 mL and the absorption was read at λ = 278 nm with a Cary 60 UV-VIS spectrophotometer equipped with a quartz cell with a path length of 1 mm. The calibration curve (Equation (1)) was prepared using the same dissolution medium (PBS) and was used to determine the concentrations of the unknown solutions:

C = A × 13.1081848, R^2^ = 0.999 (1)

where C denotes concentration (µg mL^−1^) and A—absorbance.

### 4.6. Electrochemical Experiments

Since the corrosion of Mg alloys is an electrochemical process, electrochemical methods are very often used to characterize the behaviour of these alloys in different physiological environments.

Electrochemical experiments were performed using an AutoLab PGSTAT 12 EcoChemie. All electrochemical tests were made at least three times using an electrochemical cell consisting of a reference electrode (Ag/AgCl), a counter electrode (platinum mesh), and a working electrode, at 37 °C constant temperature.

To observe the potential-time behaviour of the electrodes in PBS for a short period of time (600 s), OCP measurements were performed. It was chosen to do this experiment first because it is passive and non-destructive. Basically, we measure the potential that is established between the working electrode and the electrolyte, with respect to a reference electrode, with the counter electrode being bypassed. The OCP can give indications regarding the thermodynamic tendency of the samples to participate in the electrochemical corrosion process when introduced into the electrolyte solution.

To obtain real-time information about the corrosion behaviour of the coated and uncoated Mg alloy, electrochemical impedance spectroscopy (EIS) was used. This is one of the most modern electrochemical techniques available to characterize the electrical properties of the coatings. Since the protectiveness of a polymeric coating is related to its electrical resistance, more information about the coating performance can be obtained in situ, in a non-destructive manner. Electrochemical impedance spectroscopy characterization was carried out at OCP in the frequency range between 100 kHz and 0.01 Hz with an ac voltage amplitude of ±10 mV. Recorded data are presented as both Nyquist and Bode diagrams. Because electrochemical methods are fast tools that help to evaluate the corrosion of metals and metal alloys, another technique, namely potentiodynamic polarization, has been used for this purpose. This electrochemical method can give information about the relative rates of anodic and cathodic kinetics. The results obtained by potentiodynamic polarization allow the determination of the instantaneous corrosion rate (i_corr_) and the corrosion potential (E_corr_). Also, the corrosion rate can be determined using a second method, i.e., the polarization resistance [207]. The potentiodynamic method was used to study the behaviour of the coated and uncoated Mg alloy. After 10 min from immersion, the potential was swept with a scan rate of 2 mVs^−1^ between −0.5 V to 0.5 V vs. OCP.

### 4.7. In Vitro Biological Experiments

#### 4.7.1. Extract Preparation

The extraction procedure was carried out according to ISO 10993-12 standards [96]. Prior to the extraction procedure, the Mg-based materials were measured and sterilized overnight under ultraviolet (UV) light. To obtain the extracts, the samples were immersed for 24 h at 37 °C in Dulbecco’s Modified Eagle’s Medium (DMEM, Sigma-Aldrich Co., St. Louis, MO, USA) containing 80% fetal bovine serum (FBS, Gibco, Life Technologies Corporation, Grand Island, NY, USA) and 1% antibiotic/antimycotic (10,000 units mL^−1^ penicillin and 10 mg mL^−1^ streptomycin) (Sigma-Aldrich Co. St. Louis, MO, USA) at a ratio of the surface area to the volume of the extraction media of 3 cm^2^ mL^−1^, as previously reported [208]. After the incubation period the extracts of the uncoated and coated Mg alloy were collected for further cell-based experiments and diluted (1:7) with serum free cell culture medium.

#### 4.7.2. Cell Culture

In the present study, the cellular behaviour of the murine-derived macrophages RAW 264.7 (ATCC^®^ TIB-71™, American Type Culture Collection, Manassas, VA, USA) was investigated. The cells were seeded on tissue culture polystyrene (TCPS) plates in the corresponding diluted (1:7) extraction media and incubated in a humidified atmosphere of 5% CO_2_ at 37 °C. The initial cell density was different depending on the experimental approach. For instance, to assess the cell morphology, viability and proliferation, as well as ROS release and osteoclastogenesis process, an initial cell density of 1 × 10^4^ cells cm^−2^ was used. On the other hand, for the investigation of NO and cytokine release, the RAW 264.7 cells were seeded at an initial density of 1 × 10^5^ cells cm^−2^ while for the macrophage fusion assessment, an initial density of 2.5 × 10^3^ cells cm^−2^ was used. To note, the inflammatory behaviour of the RAW 246.7 cells, was evaluated under pro-inflammatory conditions (treatment with 100 ng mL^−1^ LPS from *Escherichia coli*), unless stated otherwise. In parallel, for the inflammatory activity, RAW 264.7 cells were incubated in DMEM supplemented with 10% FBS and antibiotic/antimycotic without (TCPS (−)) and with LPS (TCPS) and these two conditions were considered the negative (TCPS (−)) and positive (TCPS) controls for inflammation, respectively. To investigate the macrophage-osteoclast differentiation, the RAW 264.7 cells were seeded in DMEM supplemented with 10% FBS and antibiotic/antimycotic without and with 50 ng mL^−1^ RANKL, conditions which were used as negative (TCPS (−)) and positive (RANKL) control samples, respectively. For each sample, all cell culture-based assays were conducted in triplicate.

#### 4.7.3. Cell Viability and Proliferation Assays

In a first set of experiments, the potential effects of the analysed extracts on macrophage viability were assessed by cell staining with the LIVE/DEAD Viability/Cytotoxicity Kit (L-3224, Molecular Probes, Eugene, OR, USA), as we previously reported [98]. Briefly, after 24 h and 72 h of culture, the samples were rinsed with PBS, then treated with calcein AM (acetoxymethyl ester)/EthD-1 (ethidium homodimer-1) for 10 min in the dark at room temperature, and further rinsed with serum-free medium and observed with an inverted fluorescent microscope (Olympus IX71, Olympus, Tokyo, Japan). Images of representative microscopic fields were captured by means of Cell F image acquiring system (Version 5.0). To assess the extracts’ capacity to sustain cell proliferation, the Cell Counting Kit-8 (CCK-8, Sigma-Aldrich Co., St. Louis, MO, USA) was used according to the manufacturer’s instructions [209] at the same incubation periods.

#### 4.7.4. Cellular Morphology and Cytoskeleton Organization Assessment

Cell attachment, spreading and morphology were assessed by fluorescent staining of the actin cytoskeleton with phalloidin conjugated with Alexa Flour 488. At the end of the incubation time point of 24 h and 72 h, the cells were washed with PBS and fixed with 4% paraformaldehyde (PFA) for 20 min, followed by permeabilization and blocking with a solution of 0.1% Triton X-100 and 2% bovine serum albumin (BSA) in PBS for 30 min at room temperature to minimize non-specific protein–protein interactions. Afterwards, the cells were washed again with PBS and incubated with Alexa Flour 488 phalloidin (20 µg mL^−1^, Invitrogen, Eugene, OR, USA) at room temperature. Finally, the nuclei were stained with 4′,6-diaminodino-2-phenylindole (DAPI, Sigma-Aldrich Co., Steinheim, Germany) for 15 min and the cells were visualized with an inverted fluorescence microscope (Olympus IX71, Olympus, Tokyo, Japan). The images were captured by means of Cell F image acquiring system (Version 5.0). The cell area was analyzed by using Image J software (Version 1.53c, National Institutes of Health, Bethesda, MD, USA). To quantify this parameter suggestive fluorescence images of actin staining, taken at 32×, were analyzed. In order to measure the spread area of each cell, the freehand selection tool was used, and the contour of each cell was tracked manually (*n* = 10).

#### 4.7.5. In Vitro Macrophage Fusion Assay

To investigate the potential of the tested samples to induce FBGC formation from the fusion of macrophages, the cells were seeded at an initial density of 2.5 × 10^3^ cells cm^−2^ in the corresponding media under pro-inflammatory conditions (treatment with 100 ng mL^−1^ LPS). The culture media were changed every 2 days. Finally, after 7 days of incubation, the cells were treated similarly to the protocol in Section 4.7.4. After staining, the cells were observed with an inverted fluorescence microscope (Olympus IX71, Olympus, Tokyo, Japan) and the images were captured using Cell F software (Version 5.0). The level of giant cell formation was quantitatively determined by counting the nuclei using the ImageJ operating system. This helped establish a “multinuclearity index” which refers to the percentage of nuclei present in multinucleated cells, exhibiting more than 3 nuclei, in ratio to the total number of nuclei in the same culture.

#### 4.7.6. Quantification of the Secreted Pro-Inflammatory Mediators

To evaluate the effect of the tested extracts on the production of the pro-inflammatory mediators, the levels of cytokines and NO released into the culture media were quantified after 24 h and 48 h of culture. Cytokine (TNF-α, IL-6, and IL-1β) release in cell culture media/extracts was evaluated using sandwich enzyme-linked immunosorbent assays (ELISA) kits, following the manufacturer’s instructions (R&D Systems, Minneapolis, MN, USA). The optical density (OD) was measured by a microplate reader (FlexStation 3 microplate reader, Molecular Devices, USA) and the cytokine concentrations (expressed in pg mL^−1^) were calculated for each cytokine according to its related standard curve. Nitric oxide (NO) production was quantified in the samples’ extraction media using a colorimetric assay, as previously reported [98]. Thus, a mix of 50 µL of culture supernatant and 50 µL of Griess reagent (Promega, Madison, WI, USA) was obtained and incubated at room temperature, in the dark, for 10 min. Then, another 10 min incubation in the dark, at room temperature took place, after adding 50 µL of 0.1% *N*-1-napthylethylenediamine dihydrochloride aqueous solution to the mix. Finally, the OD of the coloured product was measured at 550 nm by means of a microplate reader (FlexStation 3 microplate reader, Molecular Devices, USA) and the nitrite concentration was determined by extrapolating the sodium nitrite standard curve.

#### 4.7.7. Intracellular ROS Level Determination

The levels of the intracellular ROS were determined by using 2′,7′-dichlorofluorescein diacetate (DCFH-DA, Sigma-Aldrich, St. Louis, MO, USA), a cell-permeable fluorescent compound. Briefly, RAW 264.7 cells were washed with PBS and incubated for 30 min at 37 °C with a 10 μM DCFH-DA solution prepared in PBS. Subsequently, the excess dye was removed, and the macrophages were detached by scraping. The intracellular fluorescence was measured using a microplate fluorimeter (FlexStation 3 microplate reader, Molecular Devices, Silicon Valley, CA, USA) (excitation wavelength = 488 nm and emission wavelength = 515 nm). The results were expressed as relative fluorescence units (RFU) after fluorescence intensity and reported to the number of viable cells.

#### 4.7.8. Osteoclast Differentiation Assay

The RAW 264.7 cells were seeded at an initial density of 1 × 10^4^ cells cm^−2^ and cultured in culture media/extracts supplemented with 50 ng mL^−1^ mouse recombinant RANKL (Sigma-Aldrich Co., Steinheim, Germany). The cell culture/diluted extract was changed every 2 days until osteoclasts formation was observed (7 days). The expression of TRAP was investigated by immunofluorescence staining as previously reported [210]. Briefly, after 7 days in culture, the cells were washed with PBS and, subsequently, fixed with 4% PFA. Further on, the cell membranes were permeabilized and blocked using a 0.1% Triton X-100/2% BSA solution in PBS. Afterwards, the samples were firstly incubated in a 1.2% BSA in PBS solution containing a rabbit anti-mouse TRAP antibody (Santa Cruz Biotechnology, Dallas, TX, USA), followed by a second incubation in a 1.2% BSA in PBS solution containing Alexa Flour 568-conjugated goat anti-rabbit IgG antibody (Invitrogen, Eugene, OR, USA). After staining the nuclei with DAPI for 15 min, the labelled cells were observed with an inverted fluorescence microscope (Olympus IX71, Olympus, Tokyo, Japan) and representative images were obtained using a Cell F image acquiring system (Version 5.0). The number of the multinuclear TRAP-positive cells was obtained by counting in five random regions and data are presented as the number of TRAP+ mm^−2^. For the cell morphology assessment, the actin cytoskeleton and cell nuclei were stained as presented in Section 4.7.4. The cells exhibiting more than 3 nuclei were considered to be osteoclasts. The TRAP intracellular activity was evaluated at 7 days post-seeding using 4-nitrophenyl phosphate disodium hexahydrate (pNPP disodium hexahydrate; Sigma-Aldrich Co., Steinheim, Germany) as a chromogenic substrate in accordance with a previous study [210]. The enzyme activity was evaluated by measuring the OD of the reaction product at 405 nm by using the FlexStation 3 microplate reader (Molecular Devices, San Jose, CA, USA). In addition, the number of mature osteoclasts that present an actin ring was quantified by using the Cell Counter tool of the Image J software (Version 1.53c, National Institutes of Health, Bethesda, MD, USA). Ten random fields were analyzed, 10× magnification, and from each field the total number of osteoclasts and the number of actin ring positive cells were counted. For this analysis, only cells containing at least 3 nuclei were taken into consideration. The number of actin ring positive cells was calculated as a percentage of the total number of osteoclasts.

### 4.8. Statistical Analysis

To conduct the statistical analysis of the obtained results, the GraphPad Prism software (Version 6, GraphPad, San Diego, CA, USA) based on one-way ANOVA/two-way ANOVA with Tukey’s multiple comparisons test was used. The data are presented as mean ± standard deviation (SD) and the *p* values below 0.05 are considered to be statistically significant.

## 5. Conclusions

The present study investigated the macrophage inflammatory response to PCL based-coated AZ31 Mg alloy and the influence of the immune microenvironment on the osteoclastogenic process. The obtained results demonstrate that the developed fibrous coatings on the AZ31 Mg alloy exerted effects on both macrophage inflammatory activity and macrophage-osteoclast differentiation process with important implications in the implant osseointegration process. The data obtained by FTIR and XPS analysis indicate that the Mg alloy was perfectly coated with the PCL fibers loaded with CM and/or ZnO which had an important influence on tuning the release of the active ingredient. Furthermore, by comparing with the bare alloy, the results showed an increased corrosion resistance in a PBS solution for all of the polymer coated-samples, with the Mg-PCL-ZnO and Mg-PCL-CM-ZnO exhibiting the best corrosion behaviour. The in vitro results were consistent with the findings regarding the corrosion behaviour of the PCL-based coated AZ31 Mg alloy. The indirect cell culture studies evinced that the co-presence of ZnO NP in the coating (i.e., PCL-CM-ZnO and, in a lesser extent, PCL-ZnO) induced the most favourable response in terms of morphological behaviour, cell survival and proliferation and suppression of cell fusion into FBGSs. Additionally, the ROS production was significantly lower in the extraction media obtained from the PCL-coated samples in comparisons to the bare alloy. In terms of RANKL-mediated differentiation of macrophages into osteoclasts, the quantification of TRAP activity and TRAP- positive multinucleated cells showed that that the ZnO- functionalized samples elicited the lowest levels in comparison to the rest of the analysed samples. Moreover, the actin-phalloidin staining of the osteoclasts suggested that these materials were capable of inhibiting the actin ring formation.

Despite these promising results, further in vitro and in vivo investigations are necessary in order to completely understand the interplay between the immune and bone cells in the bone regeneration process.

## Figures and Tables

**Table 1:** Surface composition of AZ31 (Mg), Mg-PCL, Mg-PCL-CM, Mg-PCL-ZnO and Mg-PCL-ZnO samples.

Code	O1s (at.%)	C1s (at.%)	Mg1s (at.%)	Al2p (at.%)
Mg	46	33	19	2
Mg-PCL	22	78	−	−
Mg-PCL-CM	21	79	−	−
Mg-PCL-CM-ZnO	24	76	−	−
Mg-PCL-ZnO	23	77	−	−

**Table 2:** Kinetic corrosion parameters in PBS for the bare and coated Mg alloy.

Sample	Tafel Slope Method				Polarisation Resistance Method	
	E_corr_, V	i_corr_, μA/cm^2^	K_g_, g/m^2^h	P, mm/an	R_P_, Ω	i_corr_, μA/cm^2^
Mg	−1.61 ± 0.02	144 ± 1.45	1.639 ± 0.02	1.751 ± 0.02	195 ± 4.5	114.52 ± 1.12
Mg-PCL	−1.52 ± 0.02	63 ± 0.67	0.717 ± 0.01	0.766 ± 0.07	865 ± 7	27.61 ± 0.25
Mg-PCL-CM	−1.48 ± 0.01	36.6 ± 0.11	0.416 ± 001	0.445 ± 0.002	923 ± 6	28.03 ± 0.16
Mg-PCL-CM-ZnO	−1.46 ± 0.01	11.4 ± 0.05	0.129 ± 0.001	0.138 ± 0.001	2898 ± 8	8.21 ± 0.04
Mg-PCL-ZnO	−1.49 ± 0.01	10.5 ± 0.04	0.119 ± 0.001	0.127 ± 0.001	3327 ± 9	7.15 ± 0.03

**Table 3:** The values of the “multinuclear index” as determined by examining 10–14 microscopicfields for each sample.

100 ng/mL LPS	Total Number of Nuclei	Nuclei in Multinuclear Cells	Multinuclear Index (%)
TCPS	1110	90	8.1
Mg Alloy	343	71	21.3
Mg-PCL	774	69	8.9
Mg-PCL-ZnO	845	15	1.75
Mg-PCL-CM	790	81	10.2
Mg-PCL-CM-ZnO	869	27	3.1

**Table 4:** Chemical composition of PBS solution.

Component	NaCl	KCl	Na_2_HPO_4_	KH_2_PO_4_
**Concentration, g/L**	8	0.2	1.144	0.2

**Table 5:** Precursors for electrospun fibrous coatings on Mg alloy.

Code	Composition		
	PCL (wt.%)	CM (mg mL^−1^)	ZnO (mg mL^−1^)
Mg-PCL	14.25	−	−
Mg-PCL-CM		5.32	−
Mg-PCL-CM-ZnO		5.32	0.25
Mg-PCL-ZnO		−	0.25

**Table 6:** The variable parameters for electrospun fibrous coatings on Mg alloy.

Code	Voltage (kV)	Flow Rate (µL min^−1^)
Mg-PCL	23	8
Mg-PCL-CM	23	8
Mg-PCL-CM-ZnO	25	12
Mg-PCL-ZnO	25	10

## Data Availability

The data presented in this study are available upon request.
